# T-bet promotes potent antitumor activity of CD4^+^ CAR T cells

**DOI:** 10.1038/s41417-018-0012-7

**Published:** 2018-03-07

**Authors:** Albert T Gacerez, Charles L Sentman

**Affiliations:** Center for Synthetic Immunity and the Department of Microbiology and Immunology, The Geisel School of Medicine at Dartmouth, Lebanon, NH 03756 USA

## Abstract

Chimeric antigen receptor (CAR) therapy has shown promise against B cell malignancies in the clinic. However, limited success in patients with solid tumors has prompted the development of new CAR strategies. In this study, a B7H6-specific CAR was combined with different variants of T-bet, a transcription factor that acts as the master regulator to induce a Th1 phenotype in CD4^+^ T cells, to create more effective CAR T cells. Skewing CD4^+^ CAR T cells into a Th1 improved CAR T cell functional activity while promoting a robust proinflammatory response against B7H6-expressing tumors. The expression of T-bet with the B7H6-specific CAR in CD4^+^ T cells conferred higher expression of the CAR, elevated secretion of Th1 and proinflammatory cytokines, and improved cellular cytotoxicity against B7H6-expressing tumor cells. In vivo, CD4^+^ T cells co-expressing a B7H6-specific CAR and T-bet improved the survival of RMA-B7H6 lymphoma-bearing mice. Thus, CD4^+^ CAR T cells with increased T-bet expression have the potential to modify the tumor microenvironment and the immune response to better treat solid and hematologic cancers.

## Introduction

Chimeric antigen receptor (CAR) T cell therapy has shown great promise as one of several emerging immunotherapies to effectively treat cancers in the clinic [1–6]. CARs are created by fusing an extracellular domain, such as a single chain fragment variable (scFv), to a transmembrane domain, followed by a costimulatory signaling molecule such as 4-1BB or CD28, and a cytoplasmic domain of CD3ζ chain to provide a primary T cell activation signal. CARs allow manipulation of T cell function and activity through the synthetic receptor, while bypassing T-cell-specific activation checkpoints, such as major histocompatibility recognition and priming by antigen presenting cells to activate antigen-specific T cells.

Previous studies have focused on elucidating the effects of the costimulatory domains and how they affect CAR T cell function [7–13]. Further innovation within the field has broadened the scope of designing CAR T cells to improve safety or expand their function. One aspect of these new advancements is the co-expression of secondary immunoregulatory genes with the CAR to direct T cell function [14–16]. For example, CAR T cells co-expressing IL-12 have shown the ability to release IL-12 at the tumor site, improving antitumor activity [17–19].

T-box expressed in T cells (T-bet) is a transcription factor widely known as the master regulator for differentiating CD4^+^ T helper cells to a T helper 1 (Th1) phenotype [20]. Th1 T cells are effective mediators of antitumor activity and have been correlated with improved prognosis in patients [21, 22]. T-bet functions to upregulate Th1-specific genes and proinflammatory pathways while suppressing those involved in differentiation of CD4^+^ T cells into Th2 cells [23–28]. T-bet modulates these pathways through its T-box DNA-binding domain, regulating genes such as IFN-γ, or through complexing with other partner proteins through its transactivation domains, such as NF-ΚB or GATA-3 [28–37].

In this study, we investigated whether the overexpression of T-bet can be used to induce a Th1-like phenotype and functional response by CD4^+^ T cells co-expressing a CAR specific for B7H6. B7H6 has been reported as a tumor-restricted ligand expressed on many different tumors [38–40]. The studies demonstrated that CD4^+^ T cells expressing a B7H6-specific CAR and overexpressing T-bet can induce a potent antitumor response and promote long-term survival in vivo.

## Materials and methods

### Mice

Female C57BL/6 (B6) mice were purchased from either the National Cancer Institute or Jackson Laboratories. Mice were 7–12 weeks old at the start of experiments. All animal experiments and procedures were ethically conducted under the approval of Dartmouth College’s Institution Animal Care and Use Committee.

### Cell lines and cell culture

The murine cell lines RMA-B7H6 and B16F10-B7H6 are murine cell lines engineered to express human B7H6 and were previously generated [40]. RMA, RMA-B7H6, B16F10, and B16F10-B7H6 cell lines were transduced with a dualtropic virus containing the PPyre9-GFP fusion gene kindly provided by Yina Huang at Dartmouth Medical School (Lebanon, NH). The cell lines underwent Puromycin selection at a concentration of 2 μg/mL. The RMA and B16F10 cell lines were obtained between 2001 and 2006. The RMA cell line was obtained from Michael Bennett (UT Southwestern Medical Center), and the B16F10 cell line was obtained from Richard Barth (Geisel School of Medicine at Dartmouth). The cell lines were not recently authenticated but have been checked for mycoplasma contamination during their use for these experiments. RMA and RMA-B7H6 cells were cultured in complete RPMI media, which was made by supplementing RPMI 1640 with 10% heat-inactive fetal bovine serum, 10 mM HEPES, 0.1 mM non-essential amino acids, 1 mM sodium pyruvate, 100 U/mL penicillin, 100 μg/mL streptomycin, and 50 μM of 2-ME. B16F10 and B16F10-B7H6 were cultured in complete Dulbecco’s modified eagle’s (DMEM) media, made with DMEM with a high glucose concentration (4.5 g/L) supplemented with the same supplements as the complete RPMI 1640 media.

### Construction of B7H6-specific CAR T-bet constructs

The B7H6-specific CAR was constructed previously [40]. The mouse T-bet gene was synthesized by Genewiz (Southplainfield, NJ, USA). T-bet (STOP) was generated by mutating nucleotide 214G→T. T-bet (**∆**TBOX) was generated by deletion of T-bet nucleotides 403–978. The B7H6-specific CAR was fused to a furin cleavage site SGSG-T2A sequence followed by T-bet or other T-bet variants (T-bet (STOP) or T-bet (**∆**TBOX)), and were cloned into the pFB-neo retroviral vector. All PCR reactions were carried out by using high-fidelity DNA polymerase Phusion (New England BioLabs, Ipswich, MA, USA) and DNA primers purchased from IDT DNA (Coralville, IA).

### Retrovirus production

Eighteen hours (h) before transfection, 2.5 × 10^6^ 293T cells were plated on a 10-cm plate in 10 mL of complete DMEM media to produce ecotropic virus. Cells were then transfected with 20 μg of a B7H6-specific CAR/T-bet plasmid and 10 μg of ψeco packaging plasmid using X-fect (Clontech), according to the manufacturer’s protocol. Forty-eight hours post transfection, media was harvested and filtered through a 0.45 μM filter before storage at −80 °C. To generate stable packaging cell lines, ecotropic virus made from 293T cells was used to infect PT67 cells followed by G418 selection (1 mg/mL) for 7 days. To make stable cell lines that produce ecotropic virus, dualtropic virus generated from PT67 cells was used to infect E86 cells followed by G418 selection (1 mg/mL). Prior to transductions, virus was concentrated with PEG-IT (System Biosciences), then re-suspended to equal titers with RPMI complete media. Virus was titered using an NIH3T3 infectivity assay. Briefly, NIH3T3 cells were plated at 0.5 × 10^5^ cells/well in a 6-well plate. Cells were transduced with supernatant from virus producing cells at various dilutions, then underwent G418 selection. After 7 days, colonies were counted and viral titer (pfu) determined.

### Generating mouse CAR T cell

CD4^+^ T cells were isolated from processed C57BL/6 spleens by magnetic bead selection and typically had a purity of >90% (Miltenyi, San Diego, CA or MojoSort, Biolegend, San Diego, CA). The CD4^+^ T cells were activated with plate-bound anti-CD3 and soluble anti-CD28 antibodies at a concentration of 500 ng/mL each for 48 h at 37 °C. T cells were transduced with virus to express the B7H6-specific CAR and mouse T-bet, T-bet (STOP), or T-bet (**∆**TBOX) constructs through spin transduction, as previously described [40]. Mock T cells were generated to serve as a negative control. Mock T cells are CD4^+^ T cells which have been transduced with a pFB-neo viral vector which contains no gene construct insertion. Concanavlin A (ConA)-activated CAR T cells were transduced with B7H6-specific CAR retrovirus, as previously described [40]. CAR T cells underwent G418 selection for 3 days at 0.5 mg/mL then histopaque enriched to isolate live cells. Purified CD4^+^ T cells and ConA-stimulated T cells were cultured in complete RPMI 1640 media with 100 U/mL or 25 U/mL of rIL-2, respectively.

### Flow cytometry

Mouse CAR T cells were stained with antibodies for CD3ε (145-2c11 Biolegend), CD4 (Clone GK1.5 Biolegend), and CD8α (clone 53-6.7 Caltag) as previously described [40]. CAR surface expression was assessed with either biotinylated Protein L (catalog no. 29997 Pierce) followed by streptavidin-Alexa 633 (Life Technologies/Invitrogen) or with a F(ab’)_2_-labeled with phycoerythrin (PE) (catalog no. 115-116-146 Jackson Immuno). Expression of T-bet was assessed with a T-bet-specific antibody (clone 4B10 Biolegend), in conjunction with a FoxP3/Transcription Factor Buffer Staining Kit (ebioscience) according to the manufacturer’s protocol. Rat IgG1 (Clone RTK2071 Biolegend) was used as the isotype control for intracellular staining. Samples were analyzed on an Accuri C6 Cytometer (BD Biosciences, San Jose, CA, USA).

### In vitro cytotoxicity

Tumor cell lines of RMA, RMA-B7H6, B16F10, and B16F10-B7H6 expressing the PPyre9-GFP gene were plated at 5 × 10^3^ cells per well in a 96-well plate. RMA and B16F10 cell lines were plated in round bottom or flat bottom plates, respectively. CAR T cells were added at various T cell effector to target ratios (E:T) of 5:1, 1:1, and 0.2:1. Cells were co-cultured at 37 °C for 24 h followed by addition of 50 μL of luciferin (200 μg/mL) (Promega) and incubated at 37 °C for 30 min before analyzing luminescence.

### Production of B7H6-Fc

Human B7H6-Fc fusion sequence was constructed by fusing the human B7H6 extracellular portion (AA 1–262) with a murine IgG2a hinge-CH2-CH3 portion, then cloned into a pCMV vector. The macaque version of B7H6-Fc was constructed similarly with macaque B7H6 extracellular portion (AA 1–262). The production and purification of B7H6-Fc was performed after transfecting the B7H6-Fc plasmid into 293F cells, as previously described [40].

### Cytokine production

ELISA plates were coated with soluble human B7H6-Fc overnight. Initial concentration started with 100 ng/well followed by fivefold serial dilutions in subsequent wells. Negative control wells were coated with 100 ng of soluble macaque B7H6-Fc. The scFv of the B7H6-specific CAR (TZ47) does not bind to macaque B7H6. Plates were washed the next day with PBS, and 5 × 10^4^ CAR T cells were cultured in these wells for 24 h. Cell-free medium was collected and analyzed for IFN-γ production by ELISA.

Total of 1 × 10^5^ CAR T cells were co-cultured with RMA, RMA-B7H6, B16F10, or B16F10-B7H6 for 24 h. Cell-free medium was collected and analyzed for cytokine production. IFN-γ was analyzed with a mouse IFN-γ ELISA kit (Biolegend). Production of various cytokines was analyzed with Luminex Multiplex kits (EMD Millipore) through DartLab.

### Treatment of tumor-bearing mice with B7H6-specific CAR T cells

Total of 1 × 10^5^ RMA-B7H6 cells were injected into mice via the tail vein in 400 μL of HBSS. Seven days later, mice were treated with 2.5 × 10^6^ ConA-stimulated T cells expressing the B7H6-specific CAR combined with 5 × 10^6^ CD4^+^ T cells expressing the B7H6-specific CAR, B7H6-specific CAR/T-bet (**∆**TBOX), or Mock CD4^+^ T cells for a total of 7.5 × 10^6^ CAR T cells. Mice were monitored and euthanized upon exhibiting moribund signs or at the end of the experiment.

### Experimental designs and statistical analysis

In all in vitro experiments, experimental conditions were run as biological triplicates. Error bars for all experiments represent standard deviations. For in vivo mouse studies, independent experiments consisted of six mice per experimental group and were not intentionally randomized after tumor injection. Investigators were not blinded to the group allocation. Survival data from two independent in vivo mouse studies were combined. The number of independent experiment runs pertaining to each figure is stated within the figure legends. To analyze differences in the mean fluorescence intensity (MFI) of CAR T cells expressing the B7H6-specific CAR or the B7H6-specific CAR/T-bet, paired *t* -test was used. Student’s *t* -test was used to analyze differences in the multiplex panel between CD4^+^ T cells expressing the B7H6-specific CAR or the B7H6-specific CAR/T-bet. Analysis of variance (ANOVA) was used to analyze differences between experimental groups comparing Mock CD4^+^ T cells, or CD4^+^ T cells expressing the B7H6-specific CAR, B7H6-specific CAR/T-bet, B7H6-specific CAR/T-bet (STOP), and B7H6-specific CAR/T-bet (**∆**TBOX). Post hoc analysis using the Dunnett’s multiple comparison test was used to assess significance of the B7H6-specific CAR/T-bet constructs expressed in T cells in comparison to T cells expressing the B7H6-specific CAR alone. In vitro cytotoxicity assays also used the Dunnett’s multiple comparison test but compared the B7H6-specific CAR constructs to Mock CD4^+^ T cells. Post hoc analysis using the Tukey’s multiple comparison test was used to assess significance between all experimental groups. A Kaplan–Meier plot was used to graph survival and the Log-rank Mantel–Cox test was used to assess statistical significance. All statistical analyses were run for two-sided comparisons under the assumption of a normal distribution. Statistical analysis was assessed through GraphPad Prism software (GraphPad Software, San Diego, CA, USA).

## Results

### Construction of B7H6-specific CAR/T-bet

**Fig. 1:**
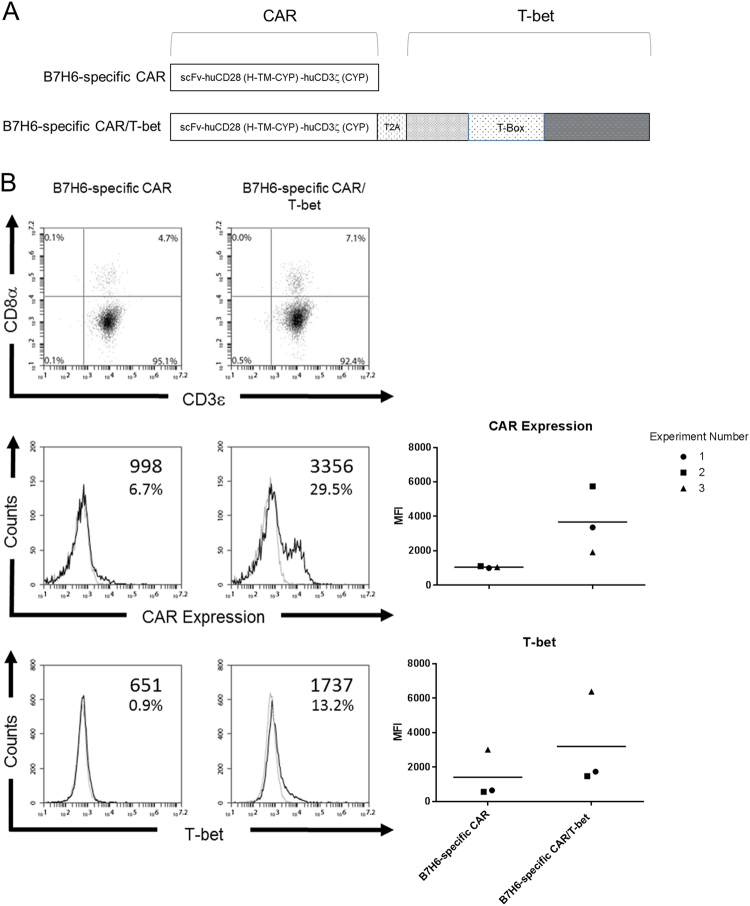
Design and expression of B7H6-specific CAR constructs. **a** The B7H6-specific CAR was created by fusing TZ47, a B7H6-specific single chain variable fragment (scFv), with the human CD28 hinge (H), transmembrane (TM), and cytoplasmic domain (CYP) followed by a human CD3ζ CYP domain. The B7H6-specific CAR/T-bet construct was created by combining the DNA of the B7H6-specific CAR with a T2A sequence followed by the mouse T-bet DNA sequence. **b** Magnetically purified mouse CD4^+^ T cells transduced with the B7H6-specific CAR or B7H6-specific CAR/T-bet were analyzed for expression of CD3, CD8, CAR expression, and T-bet. Specific antibody binding is shown in dark lines and control antibodies with light lines. Values are mean fluorescence intensity (MFI) of specific expression or percent of cells above staining control. Data shown are representative of three independent experiments. MFI and the total mean from the three independent experiments are shown (right)

To create CAR T cells that could modify the tumor microenvironment through Th1 cytokines, a CAR construct was generated that co-expressed a CAR specific for B7H6 with the transcription factor T-bet. The B7H6-specific CAR contains TZ47, a scFv specific for human B7H6, along with a human CD28 hinge, transmembrane, and cytoplasmic domains, followed by a human CD3ζ signaling domain, as previously described [40]. Co-expression of the CAR and T-bet from the same vector was achieved using a T2A containing construct. A T2A sequence was inserted between the B7H6-specific CAR construct and the T-bet transcription factor (Fig. 1a). Prior to transduction, viruses containing the CAR constructs were concentrated to an equal titer. The expression of the CAR and T-bet after transduction of purified murine CD4^+^ T cells was assessed by flow cytometry. An overall increase in both CAR expression and T-bet expression was observed in CD4^+^ T cells transduced with the B7H6-specific CAR/T-bet compared with CD4^+^ T cells transduced with the B7H6-specific CAR alone (Fig. 1b). Across three independent experiments, a general trend of increased expression of the CAR and T-bet was observed for T cells expressing B7H6-specific CAR/T-bet than the B7H6-specific CAR alone. Additionally, this increased CAR expression was also observed when total splenic T cells were used, where CD8^+^ T cells comprised the majority of the cell population (data not shown).

### T-bet overexpression in CD4^+^ CAR T cells produce more Th1 cytokines

**Fig. 2:**
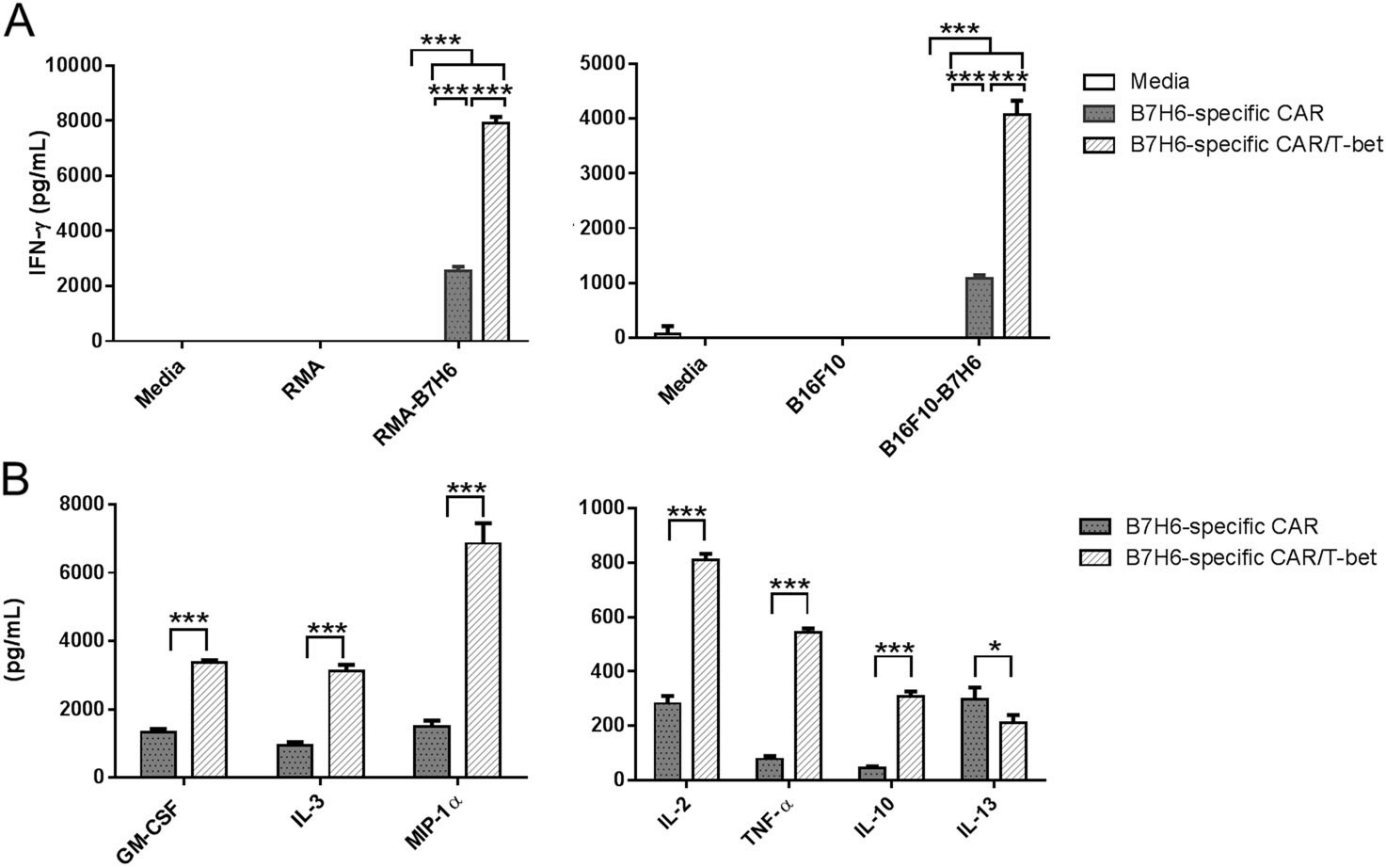
B7H6-specific CAR T cells expressing T-bet secrete different amounts of cytokine. **a** IFN-γ production by mouse CD4^+^ T cells (1 × 10^5^) expressing the B7H6-specific CAR or the B7H6-specific CAR/T-bet co-cultured with RMA tumors (1 × 10^5^), RMA-B7H6 (1 × 10^5^), B16F10 tumors (2 × 10^4^), or B16F10-B7H6 (2 × 10^4^) for 24 h. Media was used as control. ANOVA Tukey test (***p < 0.001). Data shown are representative of three independent experiments. **b** Cell-free medium from CD4^+^ T cells expressing the B7H6-specific CAR with or without T-bet co-cultured with RMA-B7H6 for 24 h was analyzed for cytokine production. Student’s *t*test (*p < 0.05, ***p < 0.001). Data shown are representative of two independent experiments

To determine whether there were functional differences in CD4^+^ CAR T cells expressing the B7H6-specific CAR or the B7H6-specific CAR/T-bet, CD4^+^-purified transduced T cells were co-cultured with RMA or B16F10 tumor cell lines with or without B7H6 expression. Cell-free medium was harvested and analyzed for IFN-γ. CAR constructs only showed activity against RMA or B16F10 tumor cells expressing B7H6, indicating specificity of the CAR to B7H6. In the co-cultures with B7H6-expressing cell lines, an increase in the amount of secreted IFN-γ from CD4^+^ T cells expressing the B7H6-specific CAR with T-bet compared to CD4^+^ T cells expressing the B7H6-specific CAR alone was observed (Fig. 2a). To determine if overexpression of T-bet promoted differentiation of CD4^+^ T cells into a Th1-like T cell, an in-depth analysis of a panel of cytokines was performed. We observed that the CD4^+^ T cells expressing the B7H6-specific CAR/T-bet produced more GM-CSF, IL-3, MIP-1α, IL-2, TNF-α, and IL-10. Additionally, the CD4^+^ T cells expressing the B7H6-specific CAR/T-bet secreted less IL-13 than the CD4^+^ T cells expressing the B7H6-specific CAR. In summary, CD4^+^ T cells expressing the B7H6-specific CAR/T-bet secreted more proinflammatory and Th1 cytokines while secreting less Th2 cytokines compared to CD4^+^ T cells expressing the B7H6-specific CAR alone.

### T-bet does not require the T-box region to enhance CD4^+^ CAR T cells

**Fig. 3:**
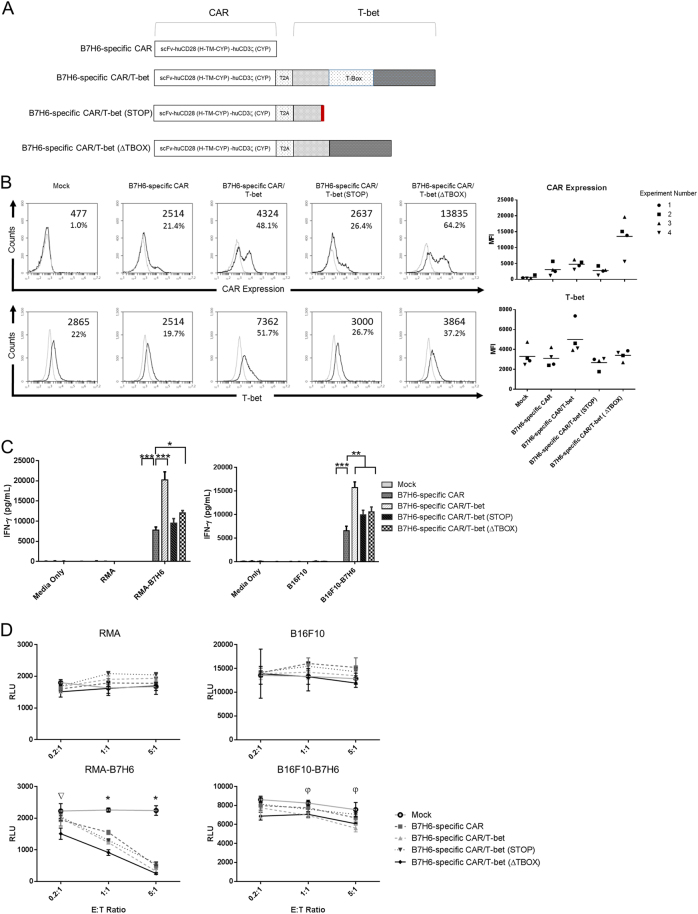
T-box domain of T-bet is not required for enhanced efficacy of CAR T cells against B7H6-expressing tumors. **a** B7H6-specific CAR/T-bet variants were constructed to remove the T-box domain from the mouse T-bet. The B7H6-specific CAR/T-bet (STOP) was created by inserting a stop codon before the T-box domain. The (**∆**TBOX) variant was created by deleting the T-box domain, leaving the transactivation domain intact. **b** Expression of mouse CD4^+^ T cells transduced with B7H6-specific CAR/T-bet variants. CD4^+^ T cells transduced with vector only (Mock) was used as a negative control. Transduced T cells were stained for T-bet or for the CAR. Specific antibody binding is shown in dark lines and fluorescence minus one (for CAR staining) or isotype control (for T-bet staining) are shown in light lines. Values indicate the mean fluorescence intensity or percent of cells above staining controls. Data shown are representative of four independent experiments. MFI and the total mean from four independent experiments are shown (right). **c** IFN-γ production by Mock CD4^+^ T cells (1 × 10^5^) or CD4^+^ T cells expressing the B7H6-specific CAR alone or with T-bet, T-bet (STOP), or T-bet (**∆**TBOX) co-cultured with RMA tumors, RMA-B7H6, B16F10 tumors, or B16F10-B7H6 for 24 h. ANOVA Dunnett’s test (*p < 0.05, **p < 0.01, ***p < 0.001). **d** Survival of tumor cells was assessed for CD4^+^ CAR T cells co-cultured with tumor cell lines at E:T ratios of 0.2:1, 1:1, and 5:1 for 24 h. The RLU values are shown ± SD. ANOVA Dunnett’s test (*p < 0.01, ^∇^*p* < 0.01 B7H6-specific CAR/TBET (**∆**TBOX) vs Mock, ^φ^*p* ≤ 0.01 B7H6-specific CAR/TBET/B7H6-specific CAR/TBET (**∆**TBOX) vs Mock). The data shown are representative of three independent experiments

To assess the extent that the T-box region was important for the effects observed with the B7H6-specific CAR with T-bet, two new constructs were generated where the mouse T-bet gene was modified to remove the T-box region (Fig. 3a). In one construct, a nonsense mutation was inserted at nucleotide position 214 to create a truncated protein, this construct was designated B7H6-specific CAR/T-bet (STOP). In the other construct, designated B7H6-specific CAR/T-bet (∆TBOX), nucleotides 403–978 were deleted, and the remaining DNA sequences were joined. This deleted the T-box domain but left the transactivation domain of T-bet intact. CD4^+^ T cells transduced with vector only served as a control. The CAR expression on CD4^+^ T cells expressing the B7H6-specific CAR/T-bet (STOP) was similar to that of CD4^+^ T cells expressing the B7H6-specific CAR but had lower expression compared to CD4^+^ T cells expressing the B7H6-specific CAR/T-bet. Interestingly, CD4^+^ T cells expressing the B7H6-specific CAR/T-bet (**∆**TBOX) construct consistently had the highest CAR expression compared to all other T cell groups (Fig. 3b). T-bet expression in CD4^+^ T cells expressing the B7H6-specific CAR/T-bet (STOP) was similar to that of the Mock control and CD4^+^ T cells expressing the B7H6-specific CAR. Generally, CD4^+^ T cells expressing the B7H6-specific CAR/T-bet (**∆**TBOX) had increased amount of T-bet compared to CD4^+^ T cells expressing the B7H6-specific CAR or the B7H6-specific CAR/T-bet (STOP) but a lower amount of T-bet compared with CD4^+^ T cells expressing the B7H6-specific CAR/T-bet.

Next, CD4^+^ T cells expressing B7H6-specific CAR/T-bet (STOP) or B7H6-specific CAR/T-bet (**∆**TBOX) were tested for differences in functional activity. CD4^+^ CAR T cells were co-cultured with either RMA or B16F10 tumor lines expressing B7H6. After 24 h, cell-free media was harvested and analyzed for secretion of IFN-γ (Fig. 3c). CD4^+^ T cells expressing the B7H6-specific CAR/T-bet (STOP), B7H6-specific CAR/T-bet (**∆**TBOX), or B7H6-specific CAR/T-bet showed an increase in the amount of secreted IFN-γ compared with the amount secretion from CD4^+^ T cells expressing the B7H6-specific CAR when co-cultured with B16F10-B7H6. In co-cultures with RMA-B7H6, increased amounts of IFN-γ were observed by CD4^+^ T cells expressing the B7H6-specific CAR/T-bet (**∆**TBOX) or the B7H6-specific CAR/T-bet compared with CD4^+^ T cells expressing only the B7H6-specific CAR. However, CD4^+^ CAR T cells expressing the B7H6-specific CAR/T-bet produced the greatest amount of IFN-γ compared to CD4^+^ T cells expressing the other CAR constructs. The data indicate that removal of the sequence coding for the T-box domain of mouse T-bet was not essential to increase CAR expression or cytokine production of CD4^+^ CAR T cells overexpressing T-bet. However, the T-box domain of T-bet may amplify effector functions of the stimulated CAR T cells.

The ability of these CD4^+^ CAR T cells to kill B7H6-expressing tumor cells was evaluated. CD4^+^ CAR T cells were co-cultured with RMA, RMA-B7H6, B16F10, or B16F10-B7H6 at various effector to target cell ratios (E:T). The tumor cells express luciferase, and a decrease in relative luminescence units (RLU) indicated tumor cell cytotoxicity. CD4^+^ CAR T cells eliminated RMA-B7H6 tumors in a robust manner compared to Mock control T cells, regardless of T-bet expression (Fig. 3d). Against B16F10-B7H6, CD4^+^ T cells expressing the B7H6-specific CAR/T-bet (**∆**TBOX) or the B7H6-specific CAR/T-bet induced cytotoxicity compared to other constructs. CD4^+^ CAR T cells co-cultured with RMA or B16F10 had comparable RLU to that of the Mock control T cells. This indicated specific killing only when B7H6 is expressed on the tumor cells. In summary, CD4^+^ CAR T cells co-expressing the B7H6-specific CAR with either T-bet or T-bet (**∆**TBOX) can effectively mediate cell killing of tumors expressing B7H6.

**Fig. 4:**
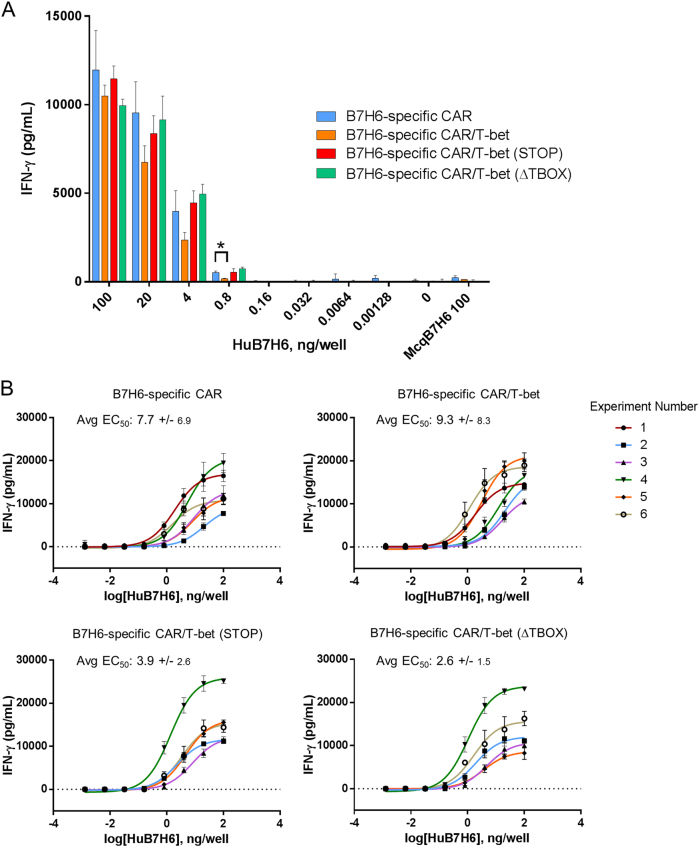
Dose stimulation of CD4^+^ CAR T cells with B7H6-Fc. **a** CD4^+^ CAR T cells with different T-bet variants were stimulated with human B7H6-Fc (HuB7H6) at various concentrations for 24 h. Cell-free medium was collected and then analyzed for IFN-γ. Macaque B7H6-Fc (McqB7H6) was used as a negative control because the TZ47 scFv does not bind McqB7H6. Data are representative of at least five independent experiments. ANOVA Tukey’s test (*p < 0.05). **b** Data from six dose stimulation experiments were plotted to make dose response curves. EC_50_ values were generated for each curve. Numbers indicate average EC_50_ values (ng/well) and standard deviations based on each CAR construct

Because differences in the amount of IFN-γ secretion among CD4^+^ T cells expressing the B7H6-specific CAR with T-bet variants were observed, we hypothesized that T-bet is specifically modulating CAR T cell function after direct CAR signaling is initiated by receptor–ligand interactions. To elucidate this, ELISA plates were coated with purified B7H6-Fc protein at various concentrations. CD4^+^ T cells expressing the different CAR constructs were cultured in these plates for 24 h, followed by analysis of the amount of IFN-γ secreted. Unexpectedly, no significant differences were observed in the amount of IFN-γ secreted by these CAR T cells at a concentration of 100 ng/well of B7H6-Fc (Fig. 4a). Data were compared across multiple experiments to identify whether the co-expression of T-bet can lower the threshold of antigen required to stimulate secretion of IFN-γ by CAR T cells. The amount of antigen needed to stimulate an IFN-γ response for all B7H6-specific CD4^+^ CAR T cells was similar, based on the average EC_50_ values of the dose response curves (Fig. 4b). Therefore, the data indicate that CD4^+^ T cells expressing T-bet or T-bet variants had no influence on modulating the response stimulated by the CAR’s interaction with the antigen alone. This suggests that T-bet may be enhancing CD4^+^ effector cell function through pathways independent of the direct signaling pathway triggered by the CAR.

### T-bet promotes survival in RMA-B7H6 lymphoma in vivo model

**Fig. 5:**
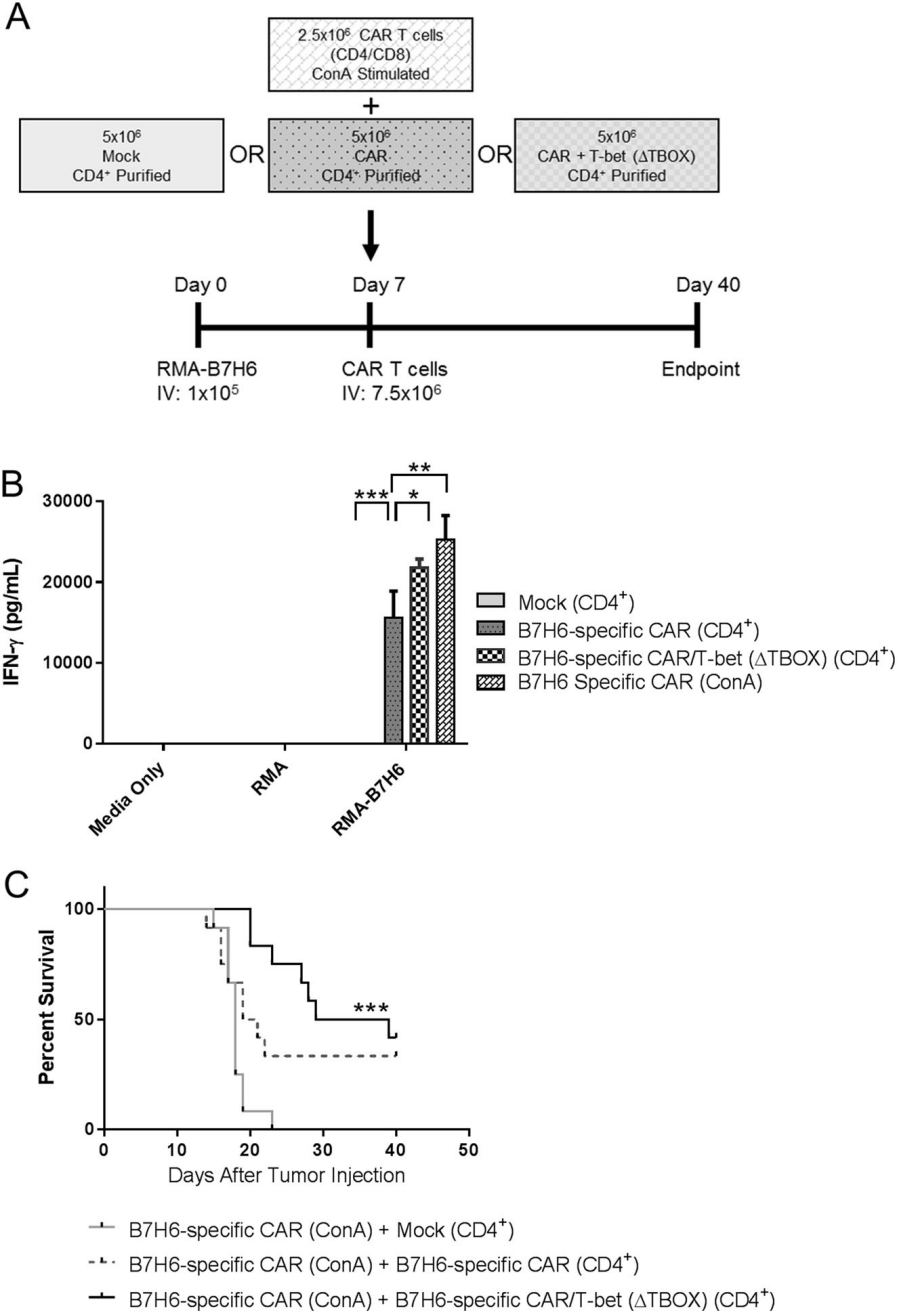
CD4^+^ CAR T cells expressing T-bet (∆TBOX) promotes survival against lymphoma-bearing mice. **a** Schematic of in vivo RMA-B7H6 lymphoma model. C57Bl/6 mice were injected with (1 × 10^5^) RMA-B7H6 intravenously (IV). Mice were then administered transduced T cells IV, 7 days post-tumor injection. Each mouse received 2.5 × 10^6^ ConA-stimulated CAR T cells with 5 × 10^6^ CD4^+^ purified Mock, B7H6-specific CAR, or B7H6-specific CAR/T-bet (**∆**TBOX) T cells (7.5 × 10^6^ T cells total). Mice were monitored for 40 days post-tumor injection. **b** In vitro IFN-γ production of transduced T cells before pooling for experiment. CD4^+^ Mock T cells, CD4^+^ B7H6-specific CAR T cells, CD4^+^ B7H6-specific CAR/T-bet (**∆**TBOX) T cells, or ConA-stimulated CAR T cells were co-cultured with RMA or RMA-B7H6 for 24 h at an E:T of 1:1. ANOVA Dunnett’s test (*p < 0.05, **p < 0.01, ***p < 0.001). The data shown are representative of two independent experiments. **c** Kaplan–Meier survival curve of RMA-B7H6 bearing mice treated with CAR T cells (*n* = 12 per group). Treatment groups as described in **a**: CD4^+^ Mock T cells + ConA-stimulated T cells, CD4^+^ B7H6-specific CAR T cells + ConA-stimulated T cells, CD4^+^ B7H6-specific CAR/T-bet (**∆**TBOX) + ConA-stimulated T cells. Data are combined from two independent experiments. Log-rank Mantel–Cox test (***p < 0.001 B7H6-specific CAR/TBET (**∆**TBOX) vs Mock)

Since CD4^+^ CAR T cells expressing the T-bet variants had enhanced functional activity against RMA-B7H6, we sought to determine whether CD4^+^ T cells co-expressing the B7H6-specific CAR/T-bet could enhance CAR T cell efficacy in vivo. The B7H6-specific CAR/T-bet (∆TBOX) construct was used to investigate the therapeutic potential in vivo as a way to study the enhanced CAR expression and function without the T-bet DNA-binding domain activity. This should mitigate any risks of having unknown gene activation by the T-box DNA-binding domain. CAR T cells were used to treat an established B7H6 lymphoma using the RMA-B7H6 lymphoma mouse model in C57BL/6 mice, which have an intact immune system. RMA-B7H6 cells express human B7H6; however, C57BL/6 mice allow these tumors to grow robustly in vivo [40]. Furthermore, previous work has shown that B7H6-specific CAR T cells originally activated with ConA can eliminate the tumors when multiple CAR T cell doses were used. These ConA CAR T cells are a mixture of ~75% CD8^+^ and 25% CD4^+^ T cells, as previously characterized [40]. To assess the impact of the CD4^+^ CAR T cells in treatment of the RMA-B7H6 model, a single dose of CAR T cells was injected, where only a partial effect of CAR T cells is expected. Along with the different CD4^+^ CAR T cells, all mice received 2.5 × 10^6^ total B7H6-specific T cells (CD8^+^ and CD4^+^ T cells), which are designated B7H6-specific CAR (ConA). Because CD8^+^ T cells have more potent cytotoxic functions compared to CD4^+^ T cells, these B7H6-specific CAR (ConA) CAR T cells provide a population that can be influenced by CD4^+^ T cells expressing a B7H6-specific CAR/T-bet construct, potentially modulating the antitumor response generated from B7H6-specific CAR (ConA) T cells in vivo. The amount of B7H6-specific CAR (ConA) T cells was reduced to provide a weak antitumor effect and provide a potential window for the CD4^+^ CAR T cells to alter survival. The experimental design for this in vivo model is depicted in Fig. 5a. T cells expressing the B7H6-specific CAR (ConA) and CD4^+^ CAR T cells were tested for functional activity against RMA-B7H6 and all T cells expressing the CAR exhibited functional activity based on the amount of IFN-γ produced (Fig. 5b). C57BL/6 mice received a mixture of B7H6-specific CAR (ConA) T cells and CD4^+^ CAR T cells with or without the T-bet (**∆**TBOX) variant 7 days after intravenous injection of RMA-B7H6. A significant improvement was observed in the overall survival of mice treated with CD4^+^ T cells expressing the B7H6 CAR-T-bet (**∆**TBOX) compared to mice treated with Mock CD4^+^ T cells (Fig. 5c). Mice treated with CD4^+^ T cells expressing the B7H6-specific CAR trended toward a significant difference compared with mice treated with Mock control CD4^+^ T cells (*p* = 0.056). Mice treated with CD4^+^ T cells expressing the B7H6-specific CAR/T-bet (**∆**TBOX) had prolonged survival with a median survival time of 34 days compared with 20 days for mice treated with CD4^+^ T cells expressing the B7H6-specific CAR. However, no difference was observed in the overall survival of mice treated with CD4^+^ CAR T cells expressing the B7H6-specific CAR compared with mice treated with CD4^+^ T cells expressing the B7H6-specific CAR/T-bet (**∆**TBOX) at this particular dose. Overall, the data indicate that CD4^+^ T cells expressing the B7H6-specific CAR/T-bet (**∆**TBOX) improved the efficacy of the CAR T cell treatment in the RMA-B7H6 lymphoma model.

## Discussion

In this study, the T-bet transcription factor was co-expressed with a CAR to augment the antitumor response generated by CD4^+^ CAR T cells. T-bet is recognized as the major transcription factor responsible for differentiating T helper CD4^+^ cells into a Th1 phenotype while simultaneously suppressing Th2 function. CD4^+^ CAR T cells overexpressing T-bet exhibit this function upon CAR stimulation, producing elevated amounts of IFN-γ, a Th1 designated cytokine directly regulated by T-bet, while reducing secretion of IL-13, a Th2 cytokine, compared to CD4^+^ T cells expressing the CAR alone. However, IL-2 secretion was increased in CAR T cells upon T-bet co-expression, challenging earlier studies that suggested T-bet inhibits IL-2 secretion [28, 34, 41, 42]. One reason for this discrepancy could be the contribution of the CAR signaling through the CD3ζ activation domain and CD28 costimulatory molecule. Activation of a T cell through the CD28 costimulatory domain has been shown to induce IL-2 secretion, which may override T-bet’s role in suppressing IL-2 production.

This study revealed that CAR expression increased when T-bet was co-expressed in CD4^+^ T cells, even though no known T-bet-binding sites are found within the construct. The increase in CAR expression on CD4^+^ T cells could affect the functional characteristics leading to enhanced cell killing or modification of the threshold for T cell activation. Altering the threshold for T cell activation may have advantages in treating tumors, such as inducing responses against tumors which express low amounts of antigen or decreasing risks of targeting normal cells expressing the antigen. Several studies on T cell stimulation through the T cell receptor (TCR) have shown that a low amount of TCR receptor expression and antigen stimulation is required to induce T cell activation [43, 44]. Additional properties, such as denser clustering of the TCR on the cell membrane or co-stimulation through CD28 signaling, have been observed to lower the activation threshold [44]. Thus, we attempted to define whether T-bet may be influencing initial stimulation of the CAR T cells by isolating the direct interactions of the CAR ligand (B7H6) with the CAR. CD4^+^ CAR T cells stimulated with antigen alone had a similar threshold for activation and produced equivalent amounts of IFN-γ, regardless of the expression of T-bet. This indicated that the expression of the B7H6-specific CAR alone on CD4^+^ T cells is sufficient to activate T cells and neither the increased T-bet expression nor the increased CAR expression observed in CD4^+^ CAR T cells expressing T-bet affected the T cell activation threshold. However, these findings did not coincide with IFN-γ secretion observed in CD4^+^ CAR T cell co-cultures with tumor cell lines expressing B7H6. Here, cell–cell interactions may enable T-bet to modulate CD4^+^ T cell functional activity and cytokine secretion.

Mutations within the T-bet gene further elucidated how the transcription factor contributes to the CD4^+^ CAR T cells phenotype and function. The T-box-binding domain is essential for allowing T-bet to directly bind DNA [35–37]. Therefore, attenuation of some of T-bet’s effect on the CD4^+^ CAR T cell was expected. This was observed with IFN-γ secretion, which is directly regulated by the binding of T-bet to the IFN-γ promoter through the T-box domain. CD4^+^ CAR T cells expressing the T-bet with a deleted T-box produced more IFN-γ than CD4^+^ T cells expressing the CAR alone, but this increase was attenuated in comparison to the amount of IFN-γ secreted by the CD4^+^ CAR T cells expressing T-bet. However, some phenotypic and functional characteristics remained intact upon mutations in T-bet. In particular, CAR expression was still upregulated in CD4^+^ CAR T cells expressing T-bet with a deleted T-box domain. This suggested that direct binding of T-bet to the DNA through the T-box domain was not required for CAR upregulation, and T-bet may be interacting with other partner proteins via the transactivation domains of T-bet. T-bet is known to interact with different partner proteins, such as NF-ΚB and GATA-3, through its transactivation domains. This enables T-bet to regulate gene expression and cellular functions without directly binding to DNA [28, 34]. T-bet lacking the T-box domain may still complex with these other protein partners, enabling CD4^+^ CAR T cells to maintain an increased expression of the CAR. Thus, T-bet is multifaceted in its ability to regulate CD4^+^ CAR T cell characteristics and opens new avenues of research to study these underlying interactions.

Multiple treatments with B7H6-specific CAR T cells have shown efficacy in immunocompetent mice bearing RMA-B7H6 [39, 40]. However, treatment with the addition of CD4^+^ T cells expressing the B7H6-specific CAR/T-bet (**∆**TBOX) improved survival despite tumor-bearing mice being treated with a single injection of these CAR T cells. The combination treatment of the CD8^+^ T cells expressing the B7H6-specific CAR and the CD4^+^ T cells expressing the B7H6-specific CAR/T-bet (**∆**TBOX) showed an overall increase in median survival time and long-term survivors compared to the control group. This suggests that the improvement in survival was dependent upon the CD4^+^ T cells expressing the B7H6-specific CAR/T-bet (**∆**TBOX), which functions as a mediator of the immune response against the tumor. Additionally, the CD4^+^ T cells expressing the B7H6-specific CAR/T-bet (**∆**TBOX) may be aiding in eliminating the tumor through cytotoxicity mechanisms, such as FAS-FASL interactions, which RMA cell lines are susceptible.

In summary, T-bet skewed the response of CD4^+^ B7H6-specific CAR T cells to secrete more proinflammatory and Th1 cytokines, while reducing the amount of Th2 cytokines when stimulated with B7H6-expressing tumors. The T-box domain of the T-bet gene was not critical for several of the phenotypic and functional differences, rather data suggest it aids in amplifying the CAR T cells’ response which has translated into an effective response in a suboptimal treatment against a mouse lymphoma. In conclusion, CAR T cells overexpressing T-bet can be used to enhance CAR T cell therapy against cancer.
